# The effects of obesity and polycystic ovary syndrome on serum lipocalin-2 levels: a cross-sectional study

**DOI:** 10.1186/1477-7827-8-151

**Published:** 2010-12-09

**Authors:** Dimitrios Panidis, Konstantinos Tziomalos, Ekaterini Koiou, Eleni A Kandaraki, Elena Tsourdi, Dimitrios Delkos, Emmanuil Kalaitzakis, Ilias Katsikis

**Affiliations:** 1 Division of Endocrinology and Human Reproduction, Second Department of Obstetrics and Gynecology, Aristotle University of Thessaloniki, Hippokration Hospital, Thessaloniki, Greece; 2 First Propedeutic Department of Internal Medicine, Aristotle University of Thessaloniki, AHEPA Hospital, Thessaloniki, Greece

## Abstract

**Background:**

Lipocalin-2 is a novel adipokine that appears to play a role in the development of insulin resistance. Serum lipocalin-2 levels are elevated in obese patients. Obesity and insulin resistance are cardinal characteristics of the polycystic ovary syndrome (PCOS). However, there are limited data on serum lipocalin-2 levels in patients with PCOS. The aim of the present study was to assess serum lipocalin-2 levels in PCOS.

**Methods:**

We studied 200 patients with PCOS and 50 healthy female volunteers.

**Results:**

Serum lipocalin-2 levels were slightly higher in women with PCOS compared with controls (65.4 +/- 34.3 vs. 60.3 +/- 26.0 ng/ml, respectively) but this difference did not reach statistical significance. In contrast, lipocalin-2 levels were higher in overweight/obese women with PCOS than in normal weight women with the syndrome (76.2 +/- 37.3 vs. 54.5 +/- 27.2 ng/ml, respectively; p < 0.001). Serum lipocalin-2 levels were also higher in overweight/obese controls compared with normal weight controls (70.1 +/- 24.9 vs. 50.5 +/- 23.7 ng/ml, respectively; p = 0.004). In the total study population (patients with PCOS and controls), lipocalin-2 levels were independently correlated with the body mass index (p < 0.001). In women with PCOS, lipocalin-2 levels were independently correlated with the waist (p < 0.001).

**Conclusions:**

Obesity is associated with elevated serum lipocalin-2 levels. In contrast, PCOS does not appear to affect lipocalin-2 levels.

## Background

Polycystic ovary syndrome (PCOS) is characterized by hyperandrogenism (biochemical hyperandrogenemia and/or clinical manifestations of hyperandrogenemia), chronic oligo- or anovulation and polycystic ovaries on ultrasonography [1,2]. Obesity, usually of the central type, is included in the cardinal characteristics of the syndrome, as it is present in varying degrees (30-70%) and is directly linked to increased peripheral insulin resistance (IR)[3-5].

Insulin resistance, via the resulting hyperinsulinemia, significantly contributes to the endocrine and metabolic disturbances observed in PCOS [6,7]. Insulin has been shown to stimulate theca cell androgen synthesis and suppress sex hormone-binding globulin (SHBG) in the liver, further increasing the free portion of circulating androgens [8,9]. In addition, adiposity contributes to the conversion of Δ_4_-androstendione (Δ_4_-A) to the most potent androgen, testosterone (T), because adipocytes have been shown to express significant amounts of the enzyme 17β-hydroxysteroid dehydrogenase-ketosteroid reductase [10,11].

Lipocalin-2 belongs to the superfamily of lipocalins and was first isolated in human neutrophils. Lipocalin-2 is a 25 kDa glucoprotein that consists of 178 aminoacid residues and is covalently linked to metalloproteinases [12,13]. The gene that encodes its synthesis is located on chromosome 9 (9q34.11) and was characterized in 1997 [14]. Lipocalin-2 mRNA has been isolated in the bone marrow, as well as in tissues exposed to microorganisms (respiratory and alimentary tract, genitourinary system). In addition, lipocalin-2 is expressed in several types of cells, including adipocytes, endothelial cells, macrophages, vascular smooth muscle cells, hepatocytes, endometrial cells and splenic cells [15-22].

Most investigators reported increased serum lipocalin-2 levels in obese patients [23,24]. In addition, males have higher serum lipocalin-2 levels and this gender difference is present in both normal weight and obese subjects [23]. Moreover, lipocalin levels are elevated in patients with cardiovascular diseases and might represent an independent cardiovascular risk factor [24].

Since a considerable proportion of patients with PCOS has obesity (particularly abdominal), IR, glucose intolerance, type 2 diabetes mellitus (T2DM) and low-grade inflammation, i.e. disorders where lipocalin-2 secretion is affected, the present study was designed to assess a) serum lipocalin-2 levels in normal weight and overweight/obese patients with PCOS, and, b) the association between serum lipocalin-2 levels and anthropometric, metabolic, hormonal and ultrasonographic features of PCOS.

## Methods

### Patients

We studied 200 women with PCOS [age 24.5 ± 5.3 years, body mass index (BMI) 27.0 ± 6.4 kg/m^2^](Group I). We also studied 50 healthy women (age 32.6 ± 4.7 years, mean BMI 25.1 ± 4.0 kg/m^2^) with normal ovulating cycles (28 ± 2 days, blood progesterone levels >10 ng/ml in two consecutive cycles), no signs of hyperandrogenism and normal sonographic appearance of the ovaries (control group, Group II)(Table 1). All women with PCOS were outpatients at the Gynecological Endocrinology Infirmary of the Second Department of Obstetrics and Gynecology, Aristotle University of Thessaloniki, Greece, who had presented with at least one of the following signs: oligomenorrhea, fertility problems, hirsutism, acne or male-pattern alopecia. Women of the control group were healthy volunteers.

**Table 1:** Anthropometric, hormonal, metabolic and ultrasonographic characteristics of all patients with polycystic ovary syndrome (PCOS) and all controls.

	Group I (patients with PCOS) (n = 200)	Group II (controls) (n = 50)	p (adjusted for age and BMI)
Age (years)	24.5 ± 5.3	32.6 ± 4.7	NA
BMI (kg/m^2^)	27.0 ± 6.4	25.1 ± 4.0	NA
Waist (cm)	83.5 ± 14.9	80.8 ± 10.1	NS
FSH (mIU/ml)	6.4 ± 1.9	7.9 ± 2.8	0.007
LH (mIU/ml)	7.6 ± 5.0	5.9 ± 2.8	NS
Prolactin (ng/ml)	14.3 ± 6.9	12.2 ± 4.3	NS
Testosterone (ng/dl)	75.1 ± 30.1	32.9 ± 14.4	< 0.001
Δ_4_-A (ng/ml)	2.9 ± 1.0	1.7 ± 0.5	< 0.001
DHEA-S (ng/ml)	3106.0 ± 1300.8	1944.6 ± 811.8	< 0.001
FAI	7.64 ± 6.1	1.98 ± 1.16	0.002
17α-OHP (ng/ml)	1.1 ± 0.5	0.7 ± 0.3	0.010
SHBG (nmol/l)	47.1 ± 28.1	69.2 ± 33.7	NS
Glucose (mg/dl)	98.5 ± 21.5	97.0 ± 9.8	NS
Insulin (μIU/ml)	12.4 ± 9.1	9.2 ± 6.8	NS
Glucose/insulin	11.52 ± 6.7	14.86 ± 9.03	NS
HOMA-IR	3.22 ± 3.90	2.24 ± 1.71	NS
QUICKI	0.34 ± 0.03	0.35 ± 0.03	NS
Area under the OGTT curve	15143.9 ± 3134.1	14565.6 ± 3352.5	NS
Mean ovarian volume (cm^3^)	9.8 ± 4.9	5.3 ± 1.8	< 0.001
Mean number of ovarian follicles	10.8 ± 4.7	6.2 ± 1.9	< 0.001
Lipocalin (ng/ml)	65.4 ± 34.3	60.3 ± 26.0	NS

NA, not applicable; NS, not significant; BMI, body mass index; FSH, follicle stimulating hormone; LH, luteinizing hormone; Δ_4_-A, Δ_4_-androstenedione; DHEA-S, dehydroepiandrosterone sulfate; FAI, free androgen index; 17α-OHP, 17α-hydroxyprogesterone; SHBG, sex hormone-binding globulin; OGTT, oral glucose tolerance test; HOMA-IR, homeostasis model assessment of insulin resistance; QUICKI, quantitative insulin sensitivity check index

Diagnosis of PCOS was based on the revised criteria of Rotterdam (see study protocol)[1,2]. None of the women studied had galactorrhea or any endocrine or systemic disease that could possibly affect reproductive physiology. No woman reported use during the last semester of any medication that could interfere with the normal function of the hypothalamic-pituitary-gonadal axis. When basic 17α-hydroxyprogesterone (17α-OHP) levels were >1.5 ng/ml, the Synacthen test (0.25 mg/1 ml; Novartis Pharma S.A., Rueil-Malmaison, France) was performed to rule out congenital adrenal hyperplasia. Other causes of hyperandrogenemia, including prolactinoma, Cushing's syndrome and androgen secreting tumors were also excluded. Informed consent was obtained from all women, and the study was approved by the institutional review board. The study met the requirements of the 1975 Helsinki guidelines.

### Study protocol

In all women, body weight, height and waist circumference (W) were measured. Body weight was measured with analog scales and in light clothing; height was measured barefoot with a stadiometer. The BMI was calculated by dividing weight (in kg) by height squared (in m) to assess obesity. The W was obtained as the smallest circumference at the level of the umbilicus.

Baseline blood samples were collected between days 3 and 7 of the menstrual cycle in the control group and between 3 to 7 days after a spontaneous bleeding episode in patients with PCOS, after an overnight fast. The circulating levels of follicle-stimulating hormone (FSH), luteinizing hormone (LH), prolactin (PRL), T, Δ_4_-A, dehydroepiandrosterone sulfate (DHEA-S), 17α-OHP, SHBG, glucose, insulin, thyroid stimulating hormone (TSH) and free thyroxin (FT4) were measured. Immediately after the baseline blood sampling an oral glucose tolerance test (OGTT) was performed; 75 g of glucose were administered orally and serum glucose levels were determined after 30, 60, 90 and 120 min. At the same day transvaginal ultrasonography was performed and the volume of each ovary was determined, as well as the number of follicles in each ovary.

Patients with PCOS were divided according to BMI in Subgroups Iα [BMI <25 kg/m^2^; n = 100, age 23.4 ± 4.5 years, BMI 22.1 ± 1.8 kg/m^2^] and Iβ (BMI >27 kg/m^2^; n = 100, age 25.7 ± 5.8 years, BMI 31.9 ± 5.6 kg/m^2^). Controls were also divided according to BMI in subgroups IIα [BMI <25 kg/m^2^; n = 25, age 31.3 ± 4.5 years, BMI 21.9 ± 1.6 kg/m^2^] and IIβ (BMI >27 kg/m^2^; n = 25, age 33.9 ± 4.6 years, BMI 28.3 ± 3.0 kg/m^2^).

## Methods

Plasma glucose, insulin, FSH, LH, PRL, androgens, 17α-OHP, TSH and FT4 concentrations were measured as previously described [25]. Serum lipocalin-2 levels were determined with an enzyme-linked immunosorbent assay (human lipocalin-2/NGAL Elisa, BioVendor Laboratorni medicina a.s., Modrice, Czech Republic). Lower levels of detection was 0.02 ng/ml, the intra-assay coefficients of variation for low and high levels were 8.38 and 7.03%, respectively, and the inter-assay coefficients of variation for low and high lipocalin-2 levels were 9.73 and 9.77%, respectively. Free androgen index (FAI) was determined as follows: FAI = T (nmol/l) × 100/SHBG (nmol/l) [26]. The homeostasis model assessment of IR (HOMA-IR) index was calculated as follows: HOMA-IR = fasting insulin (mIU/l) × glucose (mg/dl)/405 [27]. The quantitative insulin sensitivity check index (QUICKI) was calculated according to the following formula: QUICKI = 1/[log Insulin (mIU/l) + log Glucose (mg/dl))][28].

### Transvaginal ultrasonography

Transvaginal ultrasonography was performed by an experienced operator in all women. Ovarian volume was calculated as follows: Ovarian volume = (π/6) × ovarian length × ovarian height × ovarian width. Polycystic ovaries were diagnosed when ≥ 12 follicles with a diameter of 2-9 mm were present in one or both ovaries, or when the ovarian volume was > 10 cm^3^.

### Statistical analysis

Data analysis was performed with the statistical package SPSS (version 17.0; SPSS Inc., 233 South Wacker Drive, 11th Floor, Chicago, IL). All tested parameters followed normal distribution as assessed with the Kolmogorov-Smirnov test and are reported as mean ± SD. Because women with PCOS were younger and had greater BMI than controls (p <0.001 and p = 0.009, respectively), comparisons between patients and controls were performed with analysis of covariance (ANCOVA) adjusting for age and BMI. Because normal weight women with PCOS were younger than obese/overweight women with PCOS (p = 0.002), comparisons between these groups were performed with ANCOVA adjusting for age. Because normal weight controls were younger than obese/overweight controls (p = 0.046), comparisons between these groups were performed with ANCOVA adjusting for age. Changes between baseline and end-of-treatment were assessed with the paired samples t-test. Independent correlations between lipocalin-2 levels and other parameters were assessed with stepwise linear regression analysis including parameters that were significantly correlated with lipocalin-2 levels in univariate analysis. In all cases, a *p *value < 0.05 was considered significant.

## Results

The anthropometric, hormonal, metabolic and ultrasonographic features of women with PCOS and controls are shown in Table 1. Women with PCOS had lower plasma FSH levels and higher plasma T, Δ_4_-A, DHEA-S, FAI and 17α-OHP levels than controls. In addition, women with PCOS had greater mean ovarian volume and a higher mean number of ovarian follicles than controls. There were no differences in plasma glucose or insulin levels, glucose/insulin ratio, the area under the OGTT curve and the indices HOMA-IR and QUICKI between women with PCOS and controls. Serum lipocalin-2 levels were slightly higher in women with PCOS compared with controls (65.4 ± 34.3 vs. 60.3 ± 26.0 ng/ml, respectively) but this difference did not reach statistical significance.

The anthropometric, hormonal, metabolic and ultrasonographic features of normal weight and overweight/obese women with PCOS are shown in Table 2. Overweight/obese women with PCOS had greater BMI and W than normal weight women with PCOS. Plasma SHBG levels were lower and the FAI was higher in the former. Moreover, plasma insulin levels, the area under the OGTT curve and the HOMA-IR index were higher, whereas the glucose/insulin ratio and the QUICKI were lower in overweight/obese women with PCOS than in normal weight women with PCOS. Serum lipocalin-2 levels were also higher in overweight/obese women with PCOS (76.2 ± 37.3 vs. 54.5 ± 27.2 ng/ml in normal weight women with PCOS; p < 0.001).

**Table 2:** Anthropometric, hormonal, metabolic and ultrasonographic characteristics of normal weight and overweight/obese patients with polycystic ovary syndrome (PCOS).

	Group Iα (normal weight patients with PCOS) (n = 100)	Group Iβ (overweight/obese patients with PCOS) (n = 100)	p (adjusted for age)
Age (years)	23.4 ± 4.5	25.7 ± 5.8	NA
BMI (kg/m^2^)	22.1 ± 1.8	31.9 ± 5.6	< 0.001
Waist (cm)	72.8 ± 5.3	94.2 ± 13.6	< 0.001
FSH (mIU/ml)	6.8 ± 1.9	6.0 ± 1.7	0.001
LH (mIU/ml)	8.5 ± 5.5	6.7 ± 4.4	0.025
Prolactin (ng/ml)	14.7 ± 6.6	13.9 ± 7.3	NS
Testosterone (ng/dl)	74.7 ± 27.3	75.4 ± 32.7	NS
Δ_4_-A (ng/ml)	2.9 ± 1.1	2.9 ± 0.9	NS
DHEA-S (ng/ml)	3148.6 ± 1218.2	3063.1 ± 1384.1	NS
FAI	5.80 ± 4.19	9.51 ± 7.19	< 0.001
17α-OHP (ng/ml)	1.1 ± 0.5	1.1 ± 0.5	NS
SHBG (nmol/l)	59.0 ± 30.5	35.2 ± 19.4	< 0.001
Glucose (mg/dl)	94.9 ± 10.0	102.1 ± 28.3	NS
Insulin (μIU/ml)	8.6 ± 5.0	16.1 ± 10.6	< 0.001
Glucose/insulin	13.68 ± 6.02	9.36 ± 6.61	< 0.001
HOMA-IR	2.04 ± 1.34	4.38 ± 5.08	< 0.001
QUICKI	0.35 ± 0.03	0.32 ± 0.03	< 0.001
Area under the OGTT curve	14496.9 ± 2725.9	15797.4 ± 3388.2	0.006
Mean ovarian volume (cm^3^)	7.7 ± 3.7	11.9 ± 5.0	< 0.001
Mean number of ovarian follicles	10.9 ± 5.2	10.8 ± 4.1	NS
Lipocalin (ng/ml)	54.5 ± 27.2	76.2 ± 37.3	< 0.001

Abbreviations are defined in Table 1.

The anthropometric, hormonal, metabolic and ultrasonographic features of normal weight and overweight/obese controls are shown in Table 3. Overweight/obese controls had greater BMI and W than normal weight controls. Serum lipocalin-2 levels were also higher in overweight/obese controls (70.1 ± 24.9 vs. 50.5 ± 23.7 ng/ml in normal weight controls; p = 0.004). In contrast, there were no differences in hormone levels between the two groups except plasma LH levels that were higher in normal weight controls (p = 0.011). In addition, there were no differences in plasma glucose and insulin levels, the glucose/insulin ratio, the area under the OGTT curve and the indices HOMA-IR and QUICKI between normal weight controls and overweight/obese controls.

**Table 3:** Anthropometric, hormonal, metabolic and ultrasonographic characteristics of normal weight and overweight/obese controls.

	Group IIα (normal weight controls) (n = 25)	Group IIβ (overweight/obese controls) (n = 25)	p (adjusted for age)
Age (years)	31.3 ± 4.5	33.9 ± 4.6	NA
BMI (kg/m^2^)	21.9 ± 1.6	28.3 ± 3.0	< 0.001
Waist (cm)	73.7 ± 5.6	87.9 ± 8.5	< 0.001
FSH (mIU/ml)	8.1 ± 2.5	7.8 ± 3.2	NS
LH (mIU/ml)	7.1 ± 3.3	4.8 ± 1.5	0.011
Prolactin (ng/ml)	12.1 ± 3.8	12.4 ± 4.7	NS
Testosterone (ng/dl)	36.4 ± 14.5	29.4 ± 13.8	NS
Δ_4_-A (ng/ml)	1.8 ± 0.4	1.7 ± 0.6	NS
DHEA-S (ng/ml)	2138.9 ± 788.6	1750.4 ± 803.1	NS
FAI	1.89 ± 0.99	2.07 ± 1.31	NS
17α-OHP (ng/ml)	0.7 ± 0.3	0.7 ± 0.3	NS
SHBG (nmol/l)	76.4 ± 30.8	61.9 ± 35.5	NS
Glucose (mg/dl)	95.1 ± 7.8	98.9 ± 11.2	NS
Insulin (μIU/ml)	8.2 ± 7.0	10.3 ± 6.6	NS
Glucose/insulin	15.48 ± 7.27	14.23 ± 10.62	NS
HOMA-IR	1.96 ± 1.76	2.53 ± 1.63	NS
QUICKI	0.36 ± 0.03	0.35 ± 0.04	NS
Area under the OGTT curve	13009.8 ± 1680.8	16121.4 ± 3883.3	0.002
Mean ovarian volume (cm^3^)	5.3 ± 1.9	5.3 ± 1.8	NS
Mean number of ovarian follicles	6.0 ± 1.9	6.4 ± 1.9	NS
Lipocalin (ng/ml)	50.5 ± 23.7	70.1 ± 24.9	0.004

Abbreviations are defined in Table 1.

**Figure 1:**
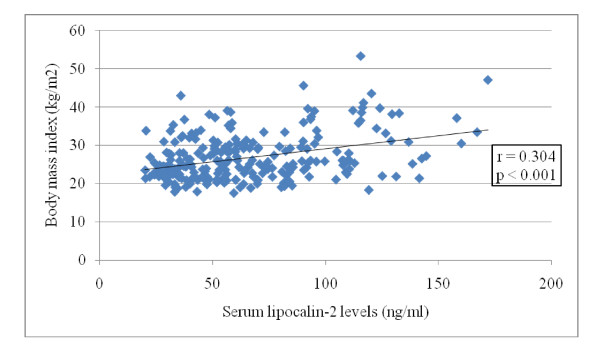
**Correlation of serum lipocalin-2 levels with the body mass index in the total study population (Groups 1 and 2, n = 250)**.

In the total sample of patients (n = 250), serum lipocalin-2 levels were negatively correlated with the QUICKI (r = -0.221, p < 0.001), the glucose/insulin ratio (r = -0.183, p = 0.004) and plasma SHBG levels (r = -0.131, p = 0.039) and positively correlated with the waist/hip ratio (r = 0.317, p < 0.001), W (r = 0.313, p < 0.001), BMI (r = 0.304, p < 0.001), HOMA-IR (r = 0.221, p < 0.001) and plasma insulin (r = 0.200, p = 0.002) and glucose levels (r = 0.191, p = 0.002). In stepwise linear regression analysis, serum lipocalin-2 levels were independently correlated with BMI (p < 0.001; Figure 1).

**Figure 2:**
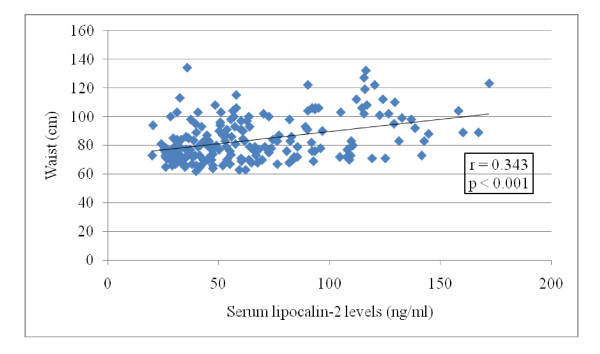
**Correlation of serum lipocalin-2 levels with the waist in women with PCOS (Group 1, n = 200)**.

In women with PCOS (n = 200), serum lipocalin-2 levels were negatively correlated with the QUICKI (r = -0.265, p < 0.001), the glucose/insulin ratio (r = -0.245, p < 0.001) and plasma SHBG levels (r = -0.152, p = 0.031) and positively correlated with the waist/hip ratio (r = 0.348, p < 0.001), W (r = 0.343, p < 0.001), BMI (r = 0.314, p < 0.001), HOMA-IR (r = 0.265, p < 0.001) and plasma insulin (r = 0.254, p < 0.001) and glucose levels (r = 0.162, p = 0.002). In stepwise linear regression analysis, serum lipocalin-2 levels were independently correlated with the W (p < 0.001; Figure 2).

## Discussion

Lipocalins are bioactive peptides that belong to adipokines. The lipocalin superfamily includes more than 20 small extracellular peptides that exert multiple functions mostly after binding to other molecules [29]. They were named lipocalins by Pervaiz and Brew from the Greek words "lipos" (i.e. fat) and "kalyx" (i.e. cup), because of their cup-like molecule [30,31]. Lipocalin-2 has a similar tertiary structure with other lipocalins and its pivotal characteristic is the presence of a hydrophobic calyx that binds to small lipophilic molecules. The main binding part of lipocalin-2 is its small iron-binding molecules [29]. Accordingly, lipocalin-2 both binds and transfers iron, an essential component for the growth of almost all bacteria. Therefore, lipocalin-2 exerts bacteriostatic actions and appears to play an important role in innate immunity and immune response to bacterial infections [32-35].

In the present study, serum lipocalin-2 levels were marginally higher in women with PCOS compared with controls (65.4 ± 34.3 vs. 60.3 ± 26.0 ng/ml, respectively) but this difference did not reach significance (Table 1). Plasma glucose or insulin levels, the glucose/insulin ratio, the area under the OGTT curve and the indices HOMA-IR and QUICKI also did not differ between women with PCOS and controls. The lack of difference in insulin resistance between patients with PCOS and controls might be partly due to the inclusion of patients with the ovulatory phenotype of PCOS, which is known to have a milder form of the metabolic disturbances [36]; among the 200 patients with PCOS, 50 (25%) had this phenotype. In addition, we used relatively insensitive markers of insulin resistance (i.e. the HOMA-IR and QUICKI indices) instead of the gold standard euglycemic hyperinsulinemic clamp and this might have also precluded the detection of a difference in insulin resistance between patients with PCOS and controls [37,38]. The slightly higher serum lipocalin-2 levels in women with PCOS (Group I) compared with controls (Group II) might be due to the greater BMI in the former (Table 1), since serum lipocalin-2 levels are elevated in obese patients [23,24]. However, the present study suggests that PCOS does not affect serum lipocalin-2 levels.

Overweight/obese women with PCOS (subgroup Iβ) had higher serum lipocalin-2 levels than normal weight women with PCOS (subgroup Iα)(p < 0.001; Table 2). Similarly, overweight/obese controls (subgroup IIβ) had higher serum lipocalin-2 levels than normal weight controls (subgroup IIα)(p = 0.004; Table 3). In the total study population (n = 250), in stepwise linear regression analysis, serum lipocalin-2 levels were independently correlated with BMI (p < 0.001; Figure 1). Moreover, in women with PCOS (n = 200), in stepwise linear regression analysis, serum lipocalin-2 levels were independently correlated with W (p < 0.001; Figure 2). A significant increase in serum lipocalin-2 levels has been previously reported in obese patients [23,24]. In addition, the elevated serum lipocalin-2 levels in obese patients correlate with anthropometric, hormonal and metabolic parameters [23]. Moreover, the strong correlation between serum lipocalin-2 levels and both the HOMA-IR index and plasma glucose levels, which is not affected after adjusting for the BMI, suggests that lipocalin-2 might represent an independent risk factor for development of IR and hyperglycemia.

There are only two studies that assessed serum lipocalin-2 levels in patients with PCOS [39,40]. However, these two studies yielded conflicting results. In the first study, serum lipocalin-2 levels were determined in 40 patients with PCOS and 40 controls, aged 25.4 ± 4.5 and 27.4 ± 4.4 years, respectively, and with BMI of 25.3 ± 3.8 and 23.4 ± 2.4 kg/m^2^, respectively [39]. The matrix metalloproteinase-9 (MMP-9)/neutrophil gelatinase-associated lipocalin (NGAL) complex was also measured. Serum lipocalin-2 and MMP-9/NGAL complex levels were lower in patients with PCOS than in controls (p < 0.001 for both comparisons)[39]. The investigators suggested that NGAL and MMP-9/NGAL complex levels should be further evaluated in patients with PCOS, because the decreased levels of these atherogenic molecules might protect patients with PCOS against cardiovascular disease (CVD). In the second study, serum lipocalin-2 levels were measured in 30 patients with PCOS and 30 controls [40]. Receiver operating characteristic curves were plotted to determine the serum levels of lipocalin-2 that indicate the presence of IR. This study showed that lipocalin-2 levels are elevated in patients with PCOS compared with controls (p < 0.001) and that lipocalin-2 may prove to be a useful marker of IR in patients with PCOS [40]. In the present study we evaluated a substantially larger number of patients with PCOS (n = 200) and we did not observe significant differences in serum lipocalin-2 levels between patients with PCOS and controls (Table 1). However, overweight/obese patients with PCOS and overweight/obese controls had significantly higher lipocalin-2 levels than normal weight patients with PCOS and normal weight controls, respectively (p < 0.001 and p = 0.004, respectively; Table 2 and 3).

It has been reported that serum lipocalin-2 levels are elevated in patients with CVD and might represent an independent cardiovascular risk factor [24]. It has also been reported that gelatinase B (also known as MMP-9), an endopeptidase capable of degrading the molecular components of the extracellular matrix, is associated with increased risk for abdominal aortic aneurysm, atherosclerosis and plaque rupture [41,42]. Therefore, MMP-9 is considered to be an important mediator of vascular remodeling and plaque instability [43]. Physical disruption of the atherosclerotic plaque triggers thrombus formation, which might lead to myocardial infarction (MI). MMP-9 action is enhanced by NGAL, also known as lipocalin-2 [44]. The formation of the MMP-9/lipocalin-2 complex is crucial for atherotic plaque erosion and thrombus formation [19,45,46]. Hemdahl et al have shown increased expression of lipocalin-2 and co-localization with MMP-9 in atherosclerotic plaques and MI lesions [47].

## Conclusions

Our findings suggest that PCOS is not associated with significant changes in serum lipocalin-2 levels. On the other hand, obese patients have elevated serum lipocalin-2 levels, regardless of the presence of PCOS. The increased serum lipocalin-2 levels in overweight and obese patients with PCOS potentially represent a useful marker of IR.

## Competing interests

The authors declare that they have no competing interests.

## Authors' contributions

DP conceived of the study, and participated in its design and coordination and drafted the manuscript. KT performed the statistical analysis and helped to draft the manuscript. All authors helped to draft the manuscript, and read and approved the final manuscript.

## References

[B1] Rotterdam ESHRE/ASRM-Sponsored PCOS Consensus Workshop Group Revised 2003 consensus on diagnostic criteria and long-term health risks related to polycystic ovary syndrome Fertil Steril 2004 81 19 25 10.1016/j.fertnstert.2003.10.004 14711538

[B2] Rotterdam ESHRE/ASRM-Sponsored PCOS Consensus Workshop Group Revised 2003 consensus on diagnostic criteria and long-term health risks related to polycystic ovary syndrome (PCOS) Hum Reprod 2004 19 41 47 10.1093/humrep/deh098 14688154

[B3] Azziz R Ehrmann D Legro RS Whitcomb RW Hanley R Fereshetian AG O'Keefe M Ghazzi MN PCOS/Troglitazone Study Group Troglitazone improves ovulation and hirsutism in the polycystic ovary syndrome: a multicenter, double blind, placebo-controlled trial J Clin Endocrinol Metab 2001 86 1626 1632 10.1210/jc.86.4.1626 11297595

[B4] Legato MJ Gender-specific aspects of obesity Int J Fertil Womens Med 1997 42 184 197 9222803

[B5] Björntorp P Abdominal obesity and the metabolic syndrome Ann Med 1992 24 465 468 1485940 10.3109/07853899209166997

[B6] Dunaif A Insulin resistance and the polycystic ovary syndrome: mechanism and implications for pathogenesis Endocr Rev 1997 18 774 800 10.1210/er.18.6.774 9408743

[B7] Goodarzi MO Korenman SG The importance of insulin resistance in polycystic ovary syndrome Fertil Steril 2003 80 255 258 10.1016/S0015-0282(03)00734-9 12909480

[B8] Nestler JE Jakubowicz DJ Lean women with polycystic ovary syndrome respond to insulin reduction with decreases in ovarian P450c17 alpha activity and serum androgens J Clin Endocrinol Metab 1997 82 4075 4079 10.1210/jc.82.12.4075 9398716

[B9] Nestler JE Powers LP Matt DW Steingold KA Plymate SR Rittmaster RS Clore JN Blackard WG A direct effect of hyperinsulinemia on serum sex hormone-binding globulin levels in obese women with the polycystic ovary syndrome J Clin Endocrinol Metab 1991 72 83 89 10.1210/jcem-72-1-83 1898744

[B10] Deslypere JP Verdonck L Vermeulen A Fat tissue: a steroid reservoir and site of steroid metabolism J Clin Endocrinol Metab 1985 61 564 570 10.1210/jcem-61-3-564 3160722

[B11] Peltoketo H Luu-The V Simard J Adamski J 17beta-hydroxysteroid dehydrogenase (HSD)/17-ketosteroid reductase (KSR) family; nomenclature and main characteristics of the 17HSD/KSR enzymes J Mol Endocrinol 1999 23 1 11 10.1677/jme.0.0230001 10431140

[B12] Triebel S Bläser J Reinke H Tschesche H A 25 kDa alpha 2-microglobulin-related protein is a component of the 125 kDa form of human gelatinase FEBS Lett 1992 314 386 388 10.1016/0014-5793(92)81511-J 1281792

[B13] Kjeldsen L Johnsen AH Sengeløv H Borregaard N Isolation and primary structure of NGAL, a novel protein associated with human neutrophil gelatinase J Biol Chem 1993 268 10425 10432 7683678

[B14] Cowland JB Borregaard N Molecular characterization and pattern of tissue expression of the gene for neutrophil gelatinase-associated lipocalin from humans Genomics 1997 45 17 23 10.1006/geno.1997.4896 9339356

[B15] Kratchmarova I Kalume DE Blagoev B Scherer PE Podtelejnikov AV Molina H Bickel PE Andersen JS Fernandez MM Bunkenborg J Roepstorff P Kristiansen K Lodish HF Mann M Pandey A A proteomic approach for identification of secreted proteins during the differentiation of 3T3-L1 preadipocytes to adipocytes Mol Cell Proteomics 2002 1 213 222 10.1074/mcp.M200006-MCP200 12096121

[B16] Liu Q Nilsen-Hamilton M Identification of a new acute phase protein J Biol Chem 1995 270 22565 22570 10.1074/jbc.270.38.22565 7545679

[B17] Meheus LA Fransen LM Raymackers JG Blockx HA Van Beeumen JJ Van Bun SM Van de Voorde A Identification by microsequencing of lipopolysaccharide-induced proteins secreted by mouse macrophages J Immunol 1993 151 1535 1547 8335946

[B18] Sunil VR Patel KJ Nilsen-Hamilton M Heck DE Laskin JD Laskin DL Acute endotoxemia is associated with upregulation of lipocalin 24p3/Lcn2 in lung and liver Exp Mol Pathol 2007 83 177 187 10.1016/j.yexmp.2007.03.004 17490638 PMC3954125

[B19] Bu DX Hemdahl AL Gabrielsen A Fuxe J Zhu C Eriksson P Yan ZQ Induction of neutrophil gelatinase-associated lipocalin in vascular injury via activation of nuclear factor-kappaB Am J Pathol 2006 169 2245 2253 10.2353/ajpath.2006.050706 17148685 PMC1762469

[B20] Jayaraman A Roberts KA Yoon J Yarmush DM Duan X Lee K Yarmush ML Identification of neutrophil gelatinase-associated lipocalin (NGAL) as a discriminatory marker of the hepatocyte-secreted protein response to IL-1beta: a proteomic analysis Biotechnol Bioeng 2005 91 502 515 10.1002/bit.20535 15918168

[B21] Huang HL Chu ST Chen YH Ovarian steroids regulate 24p3 expression in mouse uterus during the natural estrous cycle and the preimplantation period J Endocrinol 1999 162 11 19 10.1677/joe.0.1620011 10396016

[B22] Devireddy LR Teodoro JG Richard FA Green MR Induction of apoptosis by a secreted lipocalin that is transcriptionally regulated by IL-3 deprivation Science 2001 293 829 834 10.1126/science.1061075 11486081

[B23] Wang Y Lam KS Kraegen EW Sweeney G Zhang J Tso AW Chow WS Wat NM Xu JY Hoo RL Xu A Lipocalin-2 is an inflammatory marker closely associated with obesity, insulin resistance, and hyperglycemia in humans Clin Chem 2007 53 34 41 10.1373/clinchem.2006.075614 17040956

[B24] Choi KM Lee JS Kim EJ Baik SH Seo HS Choi DS Oh DJ Park CG Implication of lipocalin-2 and visfatin levels in patients with coronary heart disease Eur J Endocrinol 2008 158 203 207 10.1530/EJE-07-0633 18230827

[B25] Piouka A Farmakiotis D Katsikis I Macut D Gerou S Panidis D Anti-Müllerian hormone levels reflect severity of PCOS but are negatively influenced by obesity: relationship with increased luteinizing hormone levels Am J Physiol Endocrinol Metab 2009 296 E238 243 10.1152/ajpendo.90684.2008 18957615

[B26] Morley JE Patrick P Perry HM Evaluation of assays available to measure free testosterone Metabolism 2002 5 554 559 10.1053/meta.2002.31975 11979385

[B27] Matthews D Hosker J Rudenski A Naylor B Treacher D Turner R Homeostasis model assessment: insulin resistance and beta-cell function from fasting plasma glucose and insulin concentrations in man Diabetologia 1985 28 12 19 10.1007/BF00280883 3899825

[B28] Katz A Nambi SS Mather K Baron AD Follmann DA Sullivan G Quon MJ Quantitative insulin sensitivity check index: a simple, accurate method for assessing insulin sensitivity in humans J Clin Endocrinol Metab 2000 85 2402 2410 10.1210/jc.85.7.2402 10902785

[B29] Devarajan P Neutrophil gelatinase-associated lipocalin--an emerging troponin for kidney injury Nephrol Dial Transplant 2008 23 3737 3743 10.1093/ndt/gfn531 18809975 PMC2720816

[B30] Pervaiz S Brew K Homology of beta-lactoglobulin, serum retinol-binding protein, and protein HC Science 1985 228 335 337 10.1126/science.2580349 2580349

[B31] Pervaiz S Brew K Homology and structure-function correlations between alpha 1-acid glycoprotein and serum retinol-binding protein and its relatives FASEB J 1987 1 209 214 3622999 10.1096/fasebj.1.3.3622999

[B32] Fischbach MA Lin H Zhou L Yu Y Abergel RJ Liu DR Raymond KN Wanner BL Strong RK Walsh CT Aderem A Smith KD The pathogen-associated iroA gene cluster mediates bacterial evasion of lipocalin 2 Proc Natl Acad Sci USA 2006 103 16502 16507 10.1073/pnas.0604636103 17060628 PMC1637611

[B33] Yang J Goetz D Li JY Wang W Mori K Setlik D Du T Erdjument-Bromage H Tempst P Strong R Barasch J An iron delivery pathway mediated by a lipocalin Mol Cell 2002 10 1045 1056 10.1016/S1097-2765(02)00710-4 12453413

[B34] Flo TH Smith KD Sato S Rodriguez DJ Holmes MA Strong RK Akira S Aderem A Lipocalin 2 mediates an innate immune response to bacterial infection by sequestrating iron Nature 2004 432 917 921 10.1038/nature03104 15531878

[B35] Berger T Togawa A Duncan GS Elia AJ You-Ten A Wakeham A Fong HE Cheung CC Mak TW Lipocalin 2-deficient mice exhibit increased sensitivity to Escherichia coli infection but not to ischemia-reperfusion injury Proc Natl Acad Sci USA 2006 103 1834 1839 10.1073/pnas.0510847103 16446425 PMC1413671

[B36] Diamanti-Kandarakis E Panidis D Unravelling the phenotypic map of polycystic ovary syndrome (PCOS): a prospective study of 634 women with PCOS Clin Endocrinol 2007 67 735 742 10.1111/j.1365-2265.2007.02954.x 17760884

[B37] DeFronzo RA Tobin JD Andres R Glucose clamp technique: a method for quantifying insulin secretion and resistance Am J Physiol 1979 237 E214 E223 382871 10.1152/ajpendo.1979.237.3.E214

[B38] Diamanti-Kandarakis E Kouli C Alexandraki K Spina G Failure of mathematical indices to accurately assess insulin resistance in lean, overweight, or obese women with polycystic ovary syndrome J Clin Endocrinol Metab 2004 89 1273 1276 10.1210/jc.2003-031205 15001622

[B39] Diamanti-Kandarakis E Livadas S Kandarakis SA Margeli A Papassotiriou I Serum concentrations of atherogenic proteins neutrophil gelatinase-associated lipocalin and its complex with matrix metalloproteinase-9 are significantly lower in women with polycystic ovary syndrome: hint of a protective mechanism? Eur J Endocrinol 2008 158 525 531 10.1530/EJE-07-0822 18362300

[B40] Cakal E Ozkaya M Engin-Ustun Y Ustun Y Serum lipocalin-2 as an insulin resistance marker in patients with polycystic ovary syndrome J Endocrinol Invest 2010 e-pub ahead of print 28 May 2007; PMID: 20511727 10.1007/BF03347037 20511727

[B41] Galis ZS Sukhova GK Lark MW Libby P Increased expression of matrix metalloproteinases and matrix degrading activity in vulnerable regions of human atherosclerotic plaques J Clin Invest 1994 94 2493 2503 10.1172/JCI117619 7989608 PMC330083

[B42] Kai H Ikeda H Yasukawa H Kai M Seki Y Kuwahara F Ueno T Sugi K Imaizumi T Peripheral blood levels of matrix metalloproteases-2 and -9 are elevated in patients with acute coronary syndromes J Am Coll Cardiol 1998 32 368 372 10.1016/S0735-1097(98)00250-2 9708462

[B43] Tayebjee MH Lip GY MacFadyen RJ Matrix metalloproteinases in coronary artery disease: clinical and therapeutic implications and pathological significance Curr Med Chem 2005 12 917 925 10.2174/0929867053507270 15853705

[B44] Tong Z Wu X Ovcharenko D Zhu J Chen CS Kehrer JP Neutrophil gelatinase-associated lipocalin as a survival factor Biochem J 2005 391 441 448 10.1042/BJ20051020 16060857 PMC1276944

[B45] Yan L Borregaard N Kjeldsen L Moses MA The high molecular weight urinary matrix metalloproteinase (MMP) activity is a complex of gelatinase B/MMP-9 and neutrophil gelatinase-associated lipocalin (NGAL). Modulation of MMP-9 activity by NGAL J Biol Chem 2001 276 37258 37265 10.1074/jbc.M106089200 11486009

[B46] Leclercq A Houard X Philippe M Ollivier V Sebbag U Meilhac O Michel JB Involvement of intraplaque hemorrhage in atherothrombosis evolution via neutrophil protease enrichment J Leukoc Biol 2007 82 1420 1429 10.1189/jlb.1106671 17827339

[B47] Hemdahl AL Gabrielsen A Zhu C Eriksson P Hedin U Kastrup J Thorén P Hansson GK Expression of neutrophil gelatinase-associated lipocalin in atherosclerosis and myocardial infarction Arterioscler Thromb Vasc Biol 2006 26 136 142 10.1161/01.ATV.0000193567.88685.f4 16254208

